# Continuous process technology for bottom-up synthesis of soluble cello-oligosaccharides by immobilized cells co-expressing three saccharide phosphorylases

**DOI:** 10.1186/s12934-022-01984-1

**Published:** 2022-12-19

**Authors:** Katharina N. Schwaiger, Bernd Nidetzky

**Affiliations:** 1 acib - Austrian Centre of Industrial Biotechnology, Krenngasse 37, 8010 Graz, Austria; 2 Institute of Biotechnology and Biochemical Engineering, NAWI Graz, Graz University of Technology, Petersgasse 12, 8010, Graz, Austria

**Keywords:** Cello-oligosaccharides (COS), Multi-enzymatic cascade, Immobilized whole-cell catalyst, Polyacrylamide particles, Packed-bed reactor, Continuous process technology

## Abstract

**Background:**

Continuous processing with enzyme reuse is a well-known engineering strategy to enhance the efficiency of biocatalytic transformations for chemical synthesis. In one-pot multistep reactions, continuous processing offers the additional benefit of ensuring constant product quality via control of the product composition. Bottom-up production of cello-oligosaccharides (COS) involves multistep iterative β-1,4-glycosylation of glucose from sucrose catalyzed by sucrose phosphorylase from *Bifidobacterium adeloscentis* (BaScP), cellobiose phosphorylase from *Cellulomonas uda* (CuCbP) and cellodextrin phosphorylase from *Clostridium cellulosi* (CcCdP). Degree of polymerization (DP) control in the COS product is essential for soluble production and is implemented through balance of the oligosaccharide priming and elongation rates. A whole-cell *E. coli* catalyst co-expressing the phosphorylases in high yield and in the desired activity ratio, with CdP as the rate-limiting enzyme, was reported previously.

**Results:**

Freeze-thaw permeabilized *E. coli* cells were immobilized in polyacrylamide (PAM) at 37–111 mg dry cells/g material. PAM particles (0.25–2.00 mm size) were characterized for COS production (~ 70 g/L) in mixed vessel with catalyst recycle and packed-bed reactor set-ups. The catalyst exhibited a dry mass-based overall activity (270 U/g; 37 mg cells/g material) lowered by ~ 40% compared to the corresponding free cells due to individual enzyme activity loss, CbP in particular, caused by the immobilization. Temperature studies revealed an operational optimum at 30 °C for stable continuous reaction (~ 1 month) in the packed bed (volume: 40 mL; height: 7.5 cm). The optimum reflects the limits of PAM catalyst structural and biological stability in combination with the requirement to control COS product solubility in order to prevent clogging of the packed bed. Using an axial flow rate of 0.75 cm− 1, the COS were produced at ~ 5.7 g/day and ≥ 95% substrate conversion (sucrose 300 mM). The product stream showed a stable composition of individual oligosaccharides up to cellohexaose, with cellobiose (48 mol%) and cellotriose (31 mol%) as the major components.

**Conclusions:**

Continuous process technology for bottom-up biocatalytic production of soluble COS is demonstrated based on PAM immobilized *E. coli* cells that co-express BaScP, CuCbP and CcCdP in suitable absolute and relative activities.

**Supplementary Information:**

The online version contains supplementary material available at 10.1186/s12934-022-01984-1.

## Background

**Fig. 1:**
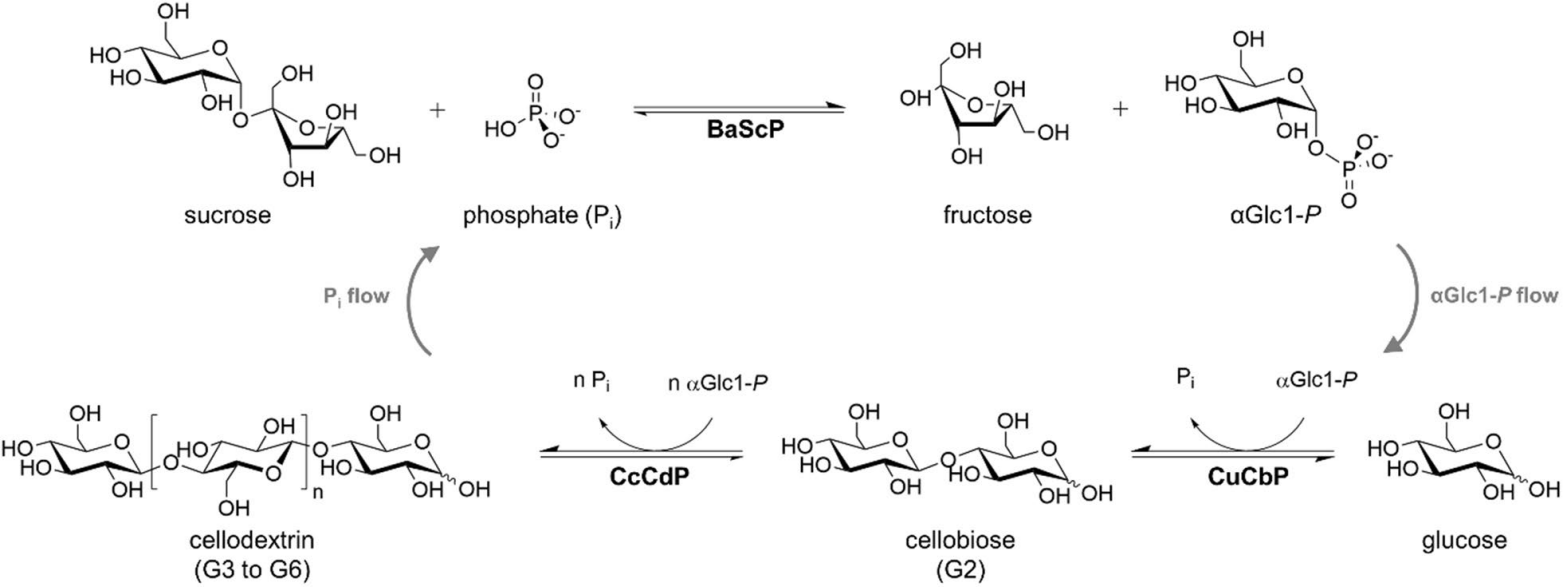
Enzymatic production of soluble COS (DP ≤ 6; G2 to G6) using sucrose phosphorylase (BaScP), cellobiose phosphorylase (CuCbP) and cellodextrin phosphorylase (CcCdP). *n* is 1 to 4

Process development in biotechnology often involves the important engineering strategy to perform the actual production, also referred to as upstream processing, continuously [1–8]. Continuous operation at steady state provides the major advantage of constancy (i.e., lack of variation with time) of key parameters of the process output (e.g., yield, product concentration, productivity) [7, 9–11]. Additionally, and this has been of decisive importance in pharma production, continuous operation facilitates consistency of the product characteristics as required by the approved product quality attributes [11–14]. Compared to pharma, quality-by-design approaches of process engineering have been less in the center of attention in the bioproduction of chemicals and ingredients [15, 16]. However, product composition represents a prime quality attribute of effectively any bioproduct irrespective of the industrial sector, the scale of production and the market price [17–24]. It is of central relevance for products that are a mixture of chemical entities. The cello-oligosaccharide (COS) product considered here is comprised of β-1,4-oligoglucose chains of variable degree of polymerization (DP) and so represents the feature of a compound mixture intrinsically. Its synthesis involves a multistep cascade transformation in one pot catalyzed by three enzymes (Fig. 1). An overall conversion of the substrate into the target product through intermediates, that are not isolated, is a hallmark of cascade biocatalysis [25–28]. Multistep biotransformations proven to be amenable for production show considerable potential to innovate organic synthesis in industrial processes [25, 29–31]. Performing such transformations continuously constitutes a promising strategy to ensure consistency in the process performance and the product(s) released [11–14].

The COS synthesis is performed through a three-enzyme phosphorylase cascade reaction in one pot, using sucrose and glucose as the substrates and phosphate as the “shuttle agent” for the glucosyl transfer (Fig. 1). α-d-Glucose 1-phosphate (αGlc1-*P*) is therefore the central intermediate from which the iterative β-1,4-glycosylation of glucose takes place. Specifically, the sucrose phosphorylase from *Bifidobacterium adolescentis* (BaScP), the cellobiose phosphorylase from *Cellulomonas uda* (CuCbP) and the cellodextrin phosphorylase from *Clostridium cellulosi* (CcCdP) are used. Overall, the immediate COS formation is controlled by two key steps, that is, priming to release the cellobiose precursor and elongation by iterative glycosylation of the cellobiose (Fig. 1) [32–35]. Both steps are fueled with αGlc1-*P* substrate by the reaction of BaScP. Given the CdP’s activity to elongate the incipient COS to a polymerization degree (DP) of only limited solubility (DP > 6), the soluble COS production necessitates an elongation rate suitably lower than the priming rate. Studies done with isolated enzymes show control of COS solubility achievable by two factors in particular [32–36]: (1) The enzyme activity ratio with BaScP in excess and CuCbP about ~ 1.5-fold higher than the limiting CcCdP [34]; and (2) the molar ratio of sucrose donor and glucose acceptor typically kept below 3 [32]. Detailed kinetic analysis of the CcCdP reaction reveals enzyme and substrate ratio as somewhat interdependent factors of reaction optimization for soluble production [33]. Moreover, the absolute elongation rate is a factor of soluble vs. insoluble COS production, dependent on the substrate concentration and the substrate ratio used [33]. In an effort at economizing the biocatalyst preparation [37, 38] for COS production, we recently developed balanced co-expression of the three phosphorylases in *Escherichia coli* [39]. The co-expression is based on a tricistron vector, referred to as pPOLY-2 (https://www.addgene.org/179276/) designed to avoid trade-off between recombinant protein production yield and activity balance. Concretely, the three phosphorylases account for ~ 46% of total intracellular *E. coli* protein [39]. The activity ratio of 10:2.9:0.6 (BaScP:CuCbP:CcCdP) reached is ideal for soluble COS production [39]. A 50 mL batch reaction that employed freeze-thaw permeabilized cells as catalyst gave 125 g/L soluble COS without the formation of insoluble COS [39]. The current study was concerned with the development of a continuous process technology based on the reported *E. coli* whole cell catalyst.

Considering that the batch-to-continuous change in mode of processing with the *E. coli* catalyst necessitates some form of immobilization of the cells [40, 41], we focused here on encapsulation in porous polyacrylamide (PAM) matrix. The idea arose from our earlier work on the development of a PAM-based whole cell BaScP catalyst for the continuous production of glucosyl glycerol in a packed-bed flow reactor [42]. To make sure, PAM is certainly not without alternatives for cell immobilization and polyvinyl alcohol (embodied in the commercialized technology of Lentikats) deserves mention in particular [43–45]. However, the PAM immobilized catalyst of whole cell BaScP involved a highly reproducible preparation and exhibited excellent in operando stability for 40 days of use in a packed-bed reactor [42]. Like in the production of COS, the substrate solution contains sucrose (a well-known microviscosogen) in high concentration. The packed bed was operated continuously without issues of overpressure or flow irregularities [42]. We therefore considered the high replication potential that a PAM-derived immobilized whole catalyst would offer for application to a continuous production of soluble COS.

We report here the preparation and detailed characterization of the PAM immobilized *E. coli* (pPOLY-2). The characterization was performed at the level of the individual phosphorylase enzymes and also overall, for COS production from sucrose and glucose. The immobilized catalyst was compared to the free cells (permeabilized with freeze-thawing) in batch and repetitive batch synthesis of COS, performed in mixed vessel reactors and with particular focus on effect of enzyme activity ratio on the distribution of individual oligosaccharides in the COS mixture. Based on evidence thus obtained, fully continuous reaction in a packed-bed reactor was developed and conditions (e.g., temperature, substrate ratio) for efficient and stable processing over several weeks were established. Overall, the here shown process technology for bottom-up biocatalytic synthesis of soluble COS appears to be promising for application to production. Soluble COS are considered as prebiotics and low calory sweeteners in feed and food [35, 46–52] and moisturizers in cosmetics [46, 53].

## Results and discussion

### Whole cell immobilization in PAM matrix

**Fig. 2:**
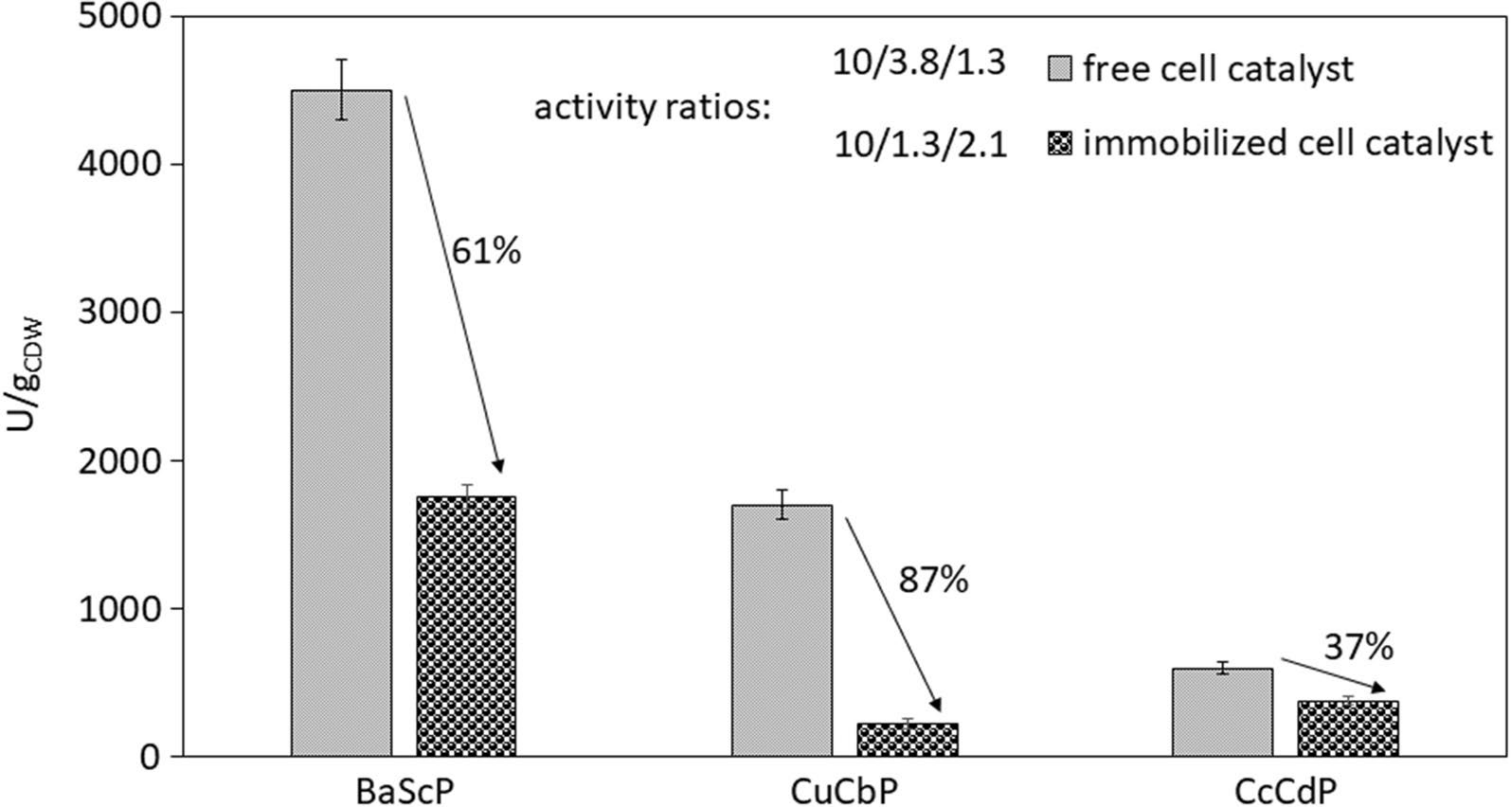
Synthesis activities at 45 °C of individual enzymes provided by the free and immobilized catalyst. One unit (U) of activity is the enzyme amount producing 1 µmol αGlc1-*P* (BaScP) or phosphate (CuCbP, CcCdP) per minute under the conditions employed. Standard deviations were calculated from three assay reactions with the same catalyst. Activity ratios were normalized to the respective BaScP activity of the catalysts

*E. coli* cells harbouring BaScP, CuCbP and CcCdP based on enzyme coexpression from the tricistron pPOLY-2 were permeabilized by freezing to − 70 °C. The thawed cells were encapsulated in PAM using a dry cell loading of 37 mg/g final material. The PAM was shredded into particles of 0.25–2.00 mm size. The particles were assayed for activity of the individual enzymes and compared to the corresponding free cells (Fig. 2). The PAM immobilization caused all activities to decrease substantially, that of CuCbP in particular. *E. coli* cells expressing the P134Q transglycosidase variant of BaScP were previously encapsulated in PAM under conditions well comparable to here [42]. Activity loss was lower considerably for the P134Q variant (≤ 30%) [42] than found here for the wild-type enzyme (~ 60%). The difference is worth noting but it was not further pursued. Differential inactivation of the individual enzymes resulted in a change in the activity ratio BaScP:CuCbP:CcCdP recorded at 45 °C from 10:3.8:1.3 in the free cells to 10:1.3:2.1 in the PAM particles. The pronounced decrease in the activity ratio CuCbP:CcCdP from 2.92 in the free cells to 0.62 in the particles is expected to cause a shift in the relative rates of oligosaccharide priming (CuCbP) and elongation (CcCdP) to favour the elongation. For the synthesis of soluble COS, as shown in earlier studies, the elongation rate should not exceed the priming rate [32–36, 39]. To counter the shortage of CuCbP activity in the immobilized cells, we used a higher molar ratio glucose:sucrose of 0.4 in the COS synthesis reactions, compared to a molar ratio of 0.3 often used in our previous works [32, 34, 39, 54]. We considered that increase in the glucose:sucrose ratio favours the priming compared to the elongation.

**Fig. 3:**
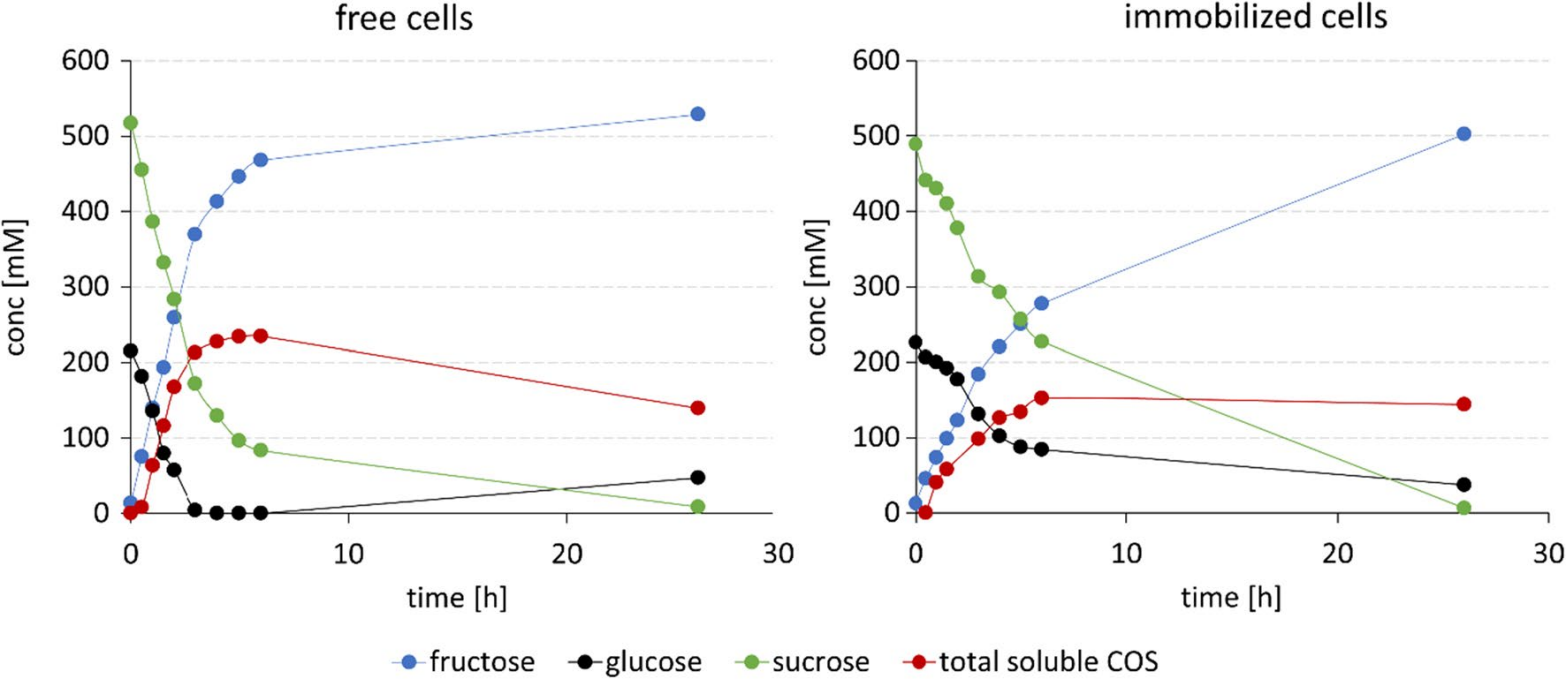
Batch reaction comparison of free and solid cell catalysts at 45 °C reaction temperature: 520 mM sucrose, 230 mM glucose, 50 mM phosphate, 50 mM MES buffer, pH 7.0, 50 mL, 3 g/L cell dry weight (CDW). Cell loading of 0.25–2.00 mm sized PAM particles was 37 mg_CDW_/g_PAM_

Time courses of soluble COS released by PAM particles and free cells are compared in Fig. 3 based on an identical volumetric loading of dry cell mass (3 g/L). The reactions were done at 50 mL volume in agitated-mixed vessel set-up. The specific catalyst activity was determined from the glucosyl units transferred into soluble COS (DP2 to DP6) in 6 h. The specific activity of the PAM particles (270 U/g dry cell mass) was ~ 57% that of the free cells. The result was consistent with the finding that CcCdP was the rate-limiting enzyme in the free cells and the decrease in the CcCdP activity by ~ 40% (Fig. 2) would thus have caused the equivalent decrease in the net COS synthesis rate. The much larger decrease in CuCbP activity (~ 87%) would still be somewhat masked in the net reaction rate. The alternative explanation, that diffusion affected the activity of the PAM particles more than it affected the activity of the free cells, cannot be excluded completely. However, given the immediate experimental evidence that decrease in the activity of an individual enzyme (CcCdP) could account for the loss of overall catalyst activity almost fully, it seemed unnecessary at this stage to explore diffusional effects. Additionally, the possible retention of larger sized COS inside the PAM particles was not pursued in these experiments. We return to these points later when discussing the results of the packed-bed reactor studies. After 26 h sucrose and glucose were converted to more than 90% in both reactions. However, due to over-elongation of soluble COS into insoluble product, the highest soluble COS accumulation was reached after 6 h with lower substrate conversions (free cells: 84/100%; immobilized cells: 57/63% for sucrose/glucose). Soluble COS concentrations of ~ 150 mM (75 g/L) and ~ 230 mM (125 g/L) were obtained in the reactions after 6 h with the PAM particles and the free cells, respectively. Insoluble product was only formed after 6 h in both reactions. The lower amount of soluble COS in the PAM particle reaction is explained by the individual activity decreases due to immobilization (Fig. 2).

**Fig. 4:**
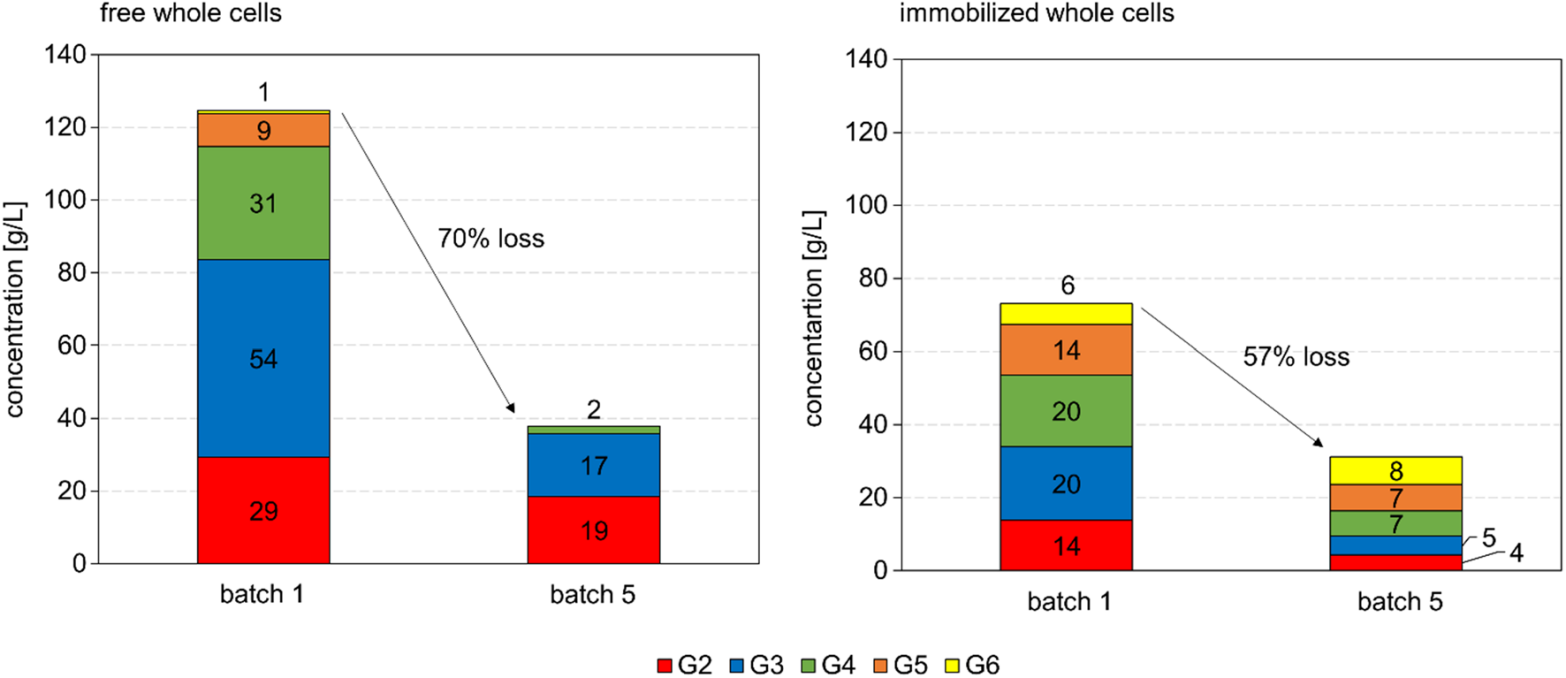
Soluble COS profile (G2 to G6 is DP2 to DP6) of product solutions after batch 1 and batch 5 (4 recycling steps). For reaction conditions see Fig. 2. Cell loading of PAM particles was 37 mg_CDW_/g_PAM_

Repetitive batch reactions with catalyst recycle were performed at 250 mL volume. Each cycle lasted 6 h and the soluble COS released at its end were measured. The total COS concentration decreased gradually from the first to the last cycle, and the COS composition also changed, as shown in Fig. 4. The PAM particle reaction yielded a more evenly distributed mixture of COS, with similar relative content of DP2 to DP5, than the free cell reaction that involved cellotriose as the major COS product (44% of total g COS). Shift in COS composition to higher DP oligosaccharides in the PAM particle reaction is consistent with the CbP:CdP activity ratio being smaller for the PAM particles than for the free cells (Fig. 2). The release of total soluble COS decreased between the first and the last cycle by 57% in the PAM particle reaction and by even 70% in the free cell reaction (Fig. 4). In the last cycle, the free cells produced cellobiose and cellotriose mainly while the PAM particles produced longer oligosaccharides (DP4 to DP6) preferentially (70% of total g COS). We determined the activity of the individual enzymes in catalyst preparations incubated under conditions identical to the batch reactions in the absence of substrate. The time used (30 h) corresponded to 5 batch cycles of 6 h each. Results in Table 1 show that activity was lost in substantial amount (≥ 55%) for each enzyme. In the PAM particles, the activity loss was most pronounced for the CuCbP (71%). Due to differential activity loss among the three enzymes, the activity ratio BaScP:CuCbP:CcCdP changed and it was 10:5.6:3.6 and 10:0.9:2 for free and immobilized cells, respectively (Table 1). Whereas in the free cells the CcCdP remained the rate-limiting enzyme after the incubation, the PAM particles featured an even stronger limitation of their overall activity by the CuCbP after the incubation than before. Results of the activity measurements were fully consistent with the observed trends in the amount and the composition of the COS products formed. They also suggested that the enzymes have become inactivated during incubation at 45 °C. The 45 °C are close to the reported temperature optima of activities for BaScP (~ 48 °C, [55]) and CuCbP (~ 45 °C, [36]). The temperature optimum of CcCdP is higher at ~ 55 °C [36]. The alternative explanation, that activity was lost due to enzyme release from the permeabilized cells, was not plausible as free and immobilized cells exhibited similar characteristics of individual enzyme stability. Enzyme release should have decreased strongly in the cells immobilized in the PAM matrix. To enhance stability, reaction at lower temperature was suggested.

**Table 1:** Synthesis activities of individual enzymes provided by the free and immobilized cell catalysts before and after incubation at 45 °C for 30 h

	Free cell catalyst					Immobilized cell catalyst				
	Before incubation		After incubation		Loss	Before incubation		After incubation		Loss
	U/g_CDW_^a^	Activity ratio	U/g_CDW_^a^	Activity ratio	%	U/g_CDW_^a^	Activity ratio	U/g_CDW_^a^	Activity ratio	%
BaScP	4500 ± 200	10	910 ± 20	10	80	1760 ± 60	10	750 ± 80	10	57
CuCbP	1700 ± 100	3.8	510 ± 80	5.6	70	224 ± 30	1.3	64 ± 10	0.9	71
CcCdP	600 ± 40	1.3	270 ± 10	2.9	55	380 ± 30	2.1	154 ± 35	2.0	59

^a^Synthesis direction at 45 °C; one unit (U) of activity is the enzyme amount producing 1 µmol αGlc1-*P* (BaScP) or phosphate (CuCbP, CcCdP) per min under the conditions employed (see “Enzyme activity measurements” section). Standard deviations were calculated from three assay reactions with the same catalyst

### Temperature effects on enzyme activity and stability, and on COS product composition

**Fig. 5:**
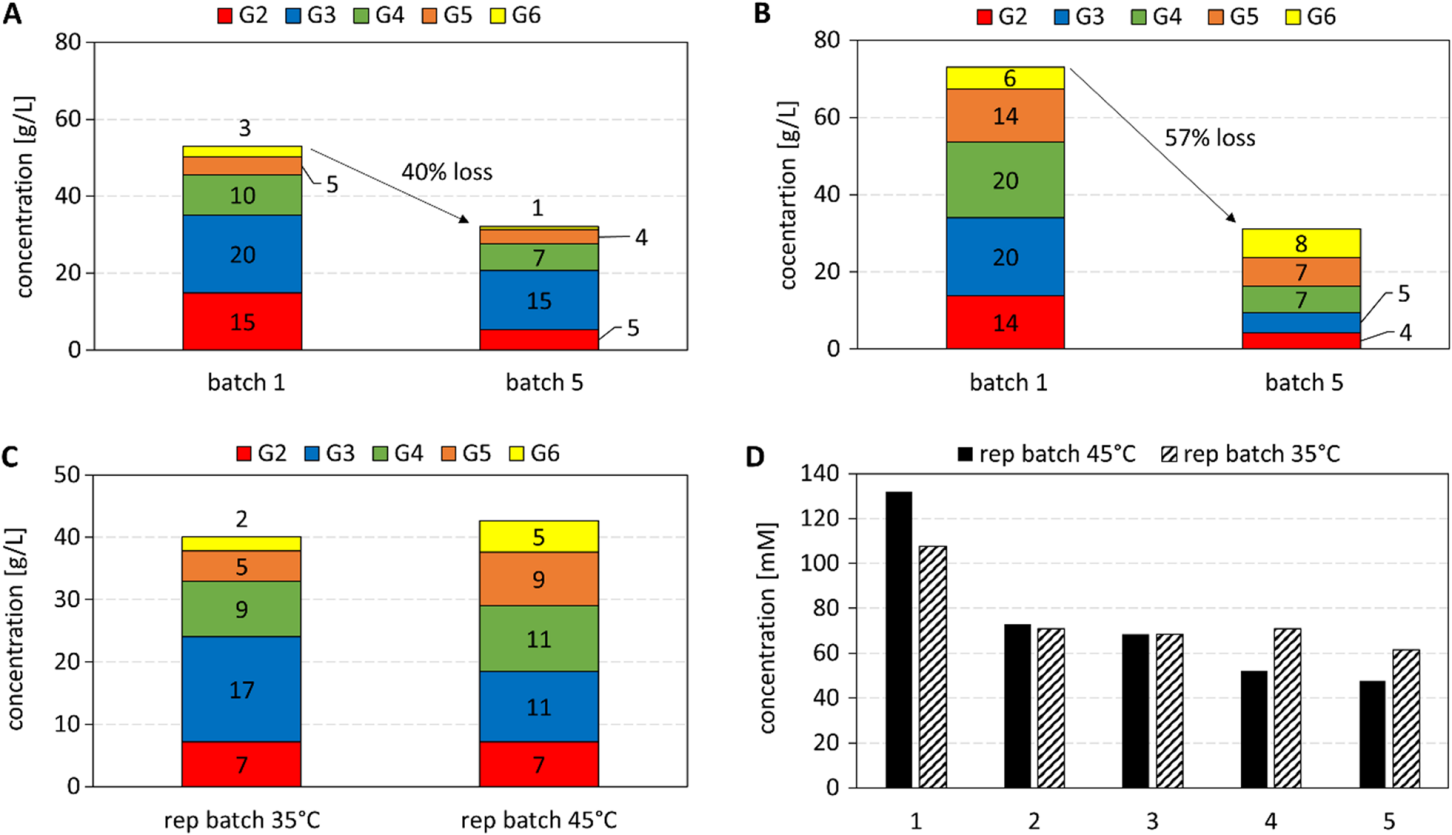
Product solutions after first and fifth batches at **A** 35 °C (8 h reaction time) and **B** 45 °C (6 h reaction time) catalyzed by immobilized cells. **C** Repetitive batches at both temperatures in a molar scale. **D** Soluble COS yield of the five batches at both temperatures

The COS synthesis by the PAM catalyst was done in repetitive batch reactions at 35 °C. Compared to 45 °C, the activity at reaction start was decreased by ~ 50% to 144 U/g cell dry mass (Additional file 1: Table S1). To account for the activity decrease, the duration of the batch was extended to 8 h. Comparison of the initial catalyst activity at the two temperatures in each cycle revealed a substantial activity loss already to the second reaction cycle and a relatively constant activity in the later cycles (Fig. 5). The activity loss to the second cycle was smaller at 35 °C (22%) compared to the 45 °C reaction (42%). At 45 °C, a further activity loss of 23% happened between the second and the last cycle. Overall, the results suggested that a substantial portion of the activity (average: 73%, ~ 105 U/g) was retained at 35 °C for at least 5 reaction cycles. The enzyme activity ratio at 35 °C was 10:1.0:1.3, corresponding to specific activities of 1050 U/g (BaScP), 100 U/g (CuCbP) and 140 U/g (CcCdP) cell dry mass.

The soluble COS concentration released at the end of each cycle at 35 °C exhibited a similar trend as the activity (Fig. 5). It dropped from a value of 108 mM in the first cycle to a relatively constant value of ~ 68 mM (average, cycle 2–5; Additional file 1: Table S1). Reaction at 45 °C also involved a large drop in soluble COS produced between the first (132 mM) and the second cycle (73 mM; Additional file 1: Table S1). Additionally, the COS concentration decreased slowly over the subsequent reaction cycles to 48 mM, lower than in the reaction at 35 °C (Additional file 1: Table S1).

Breaking down the overall decrease in the COS production into the underlying changes of individual oligosaccharides (Fig. 5 and Additional file 1: Table S1), we noted that for the reaction at 35 °C it was mostly cellobiose that was decreased in content (43 mM → 15 mM; 66%; Additional file 1: Table S1, and Fig. 5A). The COS with DP3 to DP6 exhibited a considerably smaller change. In the reaction at 45 °C, by contrast, the overall decrease in COS production was much more evenly distributed among the individual DPs (Fig. 5B). Interestingly, when the COS released in the different cycles was combined at the end of the reaction at the two temperatures, a product solution of relatively similar concentration and composition was obtained (Fig. 5C).

### Continuous COS production in a packed-bed reactor

PAM particles were assembled into a packed bed (2.6 cm inner diameter of glass column; ~ 7.5 cm height; ~ 40 mL volume) and used for COS production under varied conditions of substrate concentration, catalyst particle size, temperature and flow rate (space velocity). The results of the batch experiments provided the basis, but specific adaptations were necessary. Systematic variations were necessary to establish conditions that allowed for a stable reactor operation for weeks. The following observations were made (Table 2).

A temperature of 45 °C proved unsuitable for reason of extensive clogging of the packed bed by precipitated COS product. The effect was particle size-dependent, pronounced with large particles (3–5 mm; Additional file 1: Fig. S1A), less but still prohibitive to a stable reactor operation with particles of 0.25–2.00 mm (Additional file 1: Fig. S1B). In one run carried out with highly active particles due to maximum cell loading used (111 mg dry cell mass/g PAM), pressure increase due to clogging by the insoluble product caused the column to break (Additional file 1: Fig. S1C). The clogged bed was analyzed and the insoluble COS were found to have accumulated primarily in the interparticle space as well as on the nylon membrane at the reactor outlet. The fine-mesh nylon membrane was replaced with a paper filter of 25 μm pore size of 25 μm to prevent clogging of the reactor outlet during further experiments. However, the PAM particles had also turned white and remained so after shredding into smaller size, indicating precipitate formation in, and throughout, the particle-internal volume (Additional file 1: Fig. S1A). Additional complication of processing at 45 °C was morphological instability of the PAM particles, reflecting temperature-dependent swelling/shrinking behaviour of the PAM. An earlier study showed that PAM undergoes shrinking at a transition temperature of ~ 33 °C whereas below, it remains in a swollen state [56]. An easier and more homogeneous bed packing of our PAM catalyst was shown at 30 °C compared to 45 °C (Additional file 1: Fig. S1B). This reduced channelling of the liquid flow and overall ensured a fully reproducible process operation. The reaction at 30 °C thus suggested came with the additional benefit of an enzyme activity ratio more favourable for COS synthesis than at 45 °C. The BaScP:CuCbP:CcCdP ratio at 30 °C was determined as 10:0.6:0.7, indicating that the rates of COS priming and elongation were balanced reasonably at this temperature (Additional file 1: Table S2). As already mentioned, the CcCdP elongation activity exceeded the CuCbP priming activity ~ 1.6-fold at 45 °C (Fig. 2).

To minimize the risk of reactor clogging during continuous processing even at 30 °C, the glucose concentration was increased to 300 mM, giving a sucrose:glucose ratio of 1. Additionally, the dry cell loading into the PAM was limited to 74 mg/g (Table 2, no. 4). Higher [glucose] would shift the distribution of the oligosaccharides released to a lower DP or, in other words, towards a better solubility. The resulting composition change of the overall COS product was considered to be acceptable at this point of the development. The cell loading would delimit the volumetric oligosaccharide elongation rate by the CcCdP such that the characteristic time of the reaction, determined by the applied space velocity in the continuous experiment, could be used for convenient control of the product DP even at high degrees of substrate conversion. Applying the stated conditions, the space velocity was varied between 0.6 h^− 1^ and 0.1 h^− 1^ and the reactor left on stream for a time corresponding to 8 or more turnovers of the packed-bed volume. At each space velocity, the attainment and maintenance of a steady state was confirmed in multiple samples analyzed. Results in Table 2 show increase in the concentration of total COS released when the space velocity was decreased. The space-time yield (STY) showed reciprocal dependence (decrease) on the corresponding space velocity change, as expected. Breaking down the COS mixture into two oligosaccharide fractions G2 and G3–G6 the interesting trend is noted that the concentration of DP2 (cellobiose) increased proportionally (~ 6-fold) with the decrease in space velocity while concentration change of the G3–G6 fraction was just ~ 2–3-fold (experiments no. 4 and 5, Table 2). Challenges of analytics of the higher DP oligosaccharides are discussed later and the measurements of G3 to G6 involved larger error than measurement of G2. However, the effect of the space velocity change was significant and it indicated elongation rate less strongly dependent on the space velocity than the priming rate. Tentative explanation is that external liquid-solid mass transfer in the packed bed (enhanced by increased space velocity) affects the elongation rate more strongly than the priming rate. Since αGlc1P is required in both reactions, it might be important that the substrate of the priming reaction (glucose) was available in larger amount (~ 2-fold) than the total substrate concentration for elongation (G2 and higher). We recognize the limitation of our discussion at this stage and the effect may warrant further investigation in the future. Practically, the results suggested reactor operation at a space velocity of 0.1 h^− 1^ to achieve complete conversion of the sucrose used.

**Table 2:** Comparison of continuous production experiments with varying particle sizes, cell loadings, substrate concentrations, and space velocities

No.	Particle size [mm]	Cell loading [mg_CDW_/g_PAM_]	Sucrose/glucose [mM]	SV [h^− 1^]	T [°C]	G2 yield [mM]	G3-6 yield [mM]		STY [g/L/h]	In operando [days]	
1	3.00–5.00	37	300/200	0.1	45	50	0		1.7	2	
2	0.25–2.00	37	300/200	0.5	45	50	7		10.0	2	
3	0.25–2.00	111	300/200	0.1	30	60	91		7.6	2	
4	**0.25–2.00**	**74**	300/300	0.6	30	12	27		14	1	
			300/300	0.3	30	25	30		7.8	1	
			**300/300**	**0.1**	**30**	**66**	**49**		**5.2**	**7**	
5	**0.25–2.00**	**74**	**300/300**	**0.1**	**30**	**66**	**71**		**6.6**	**26**	

**Fig. 6:**
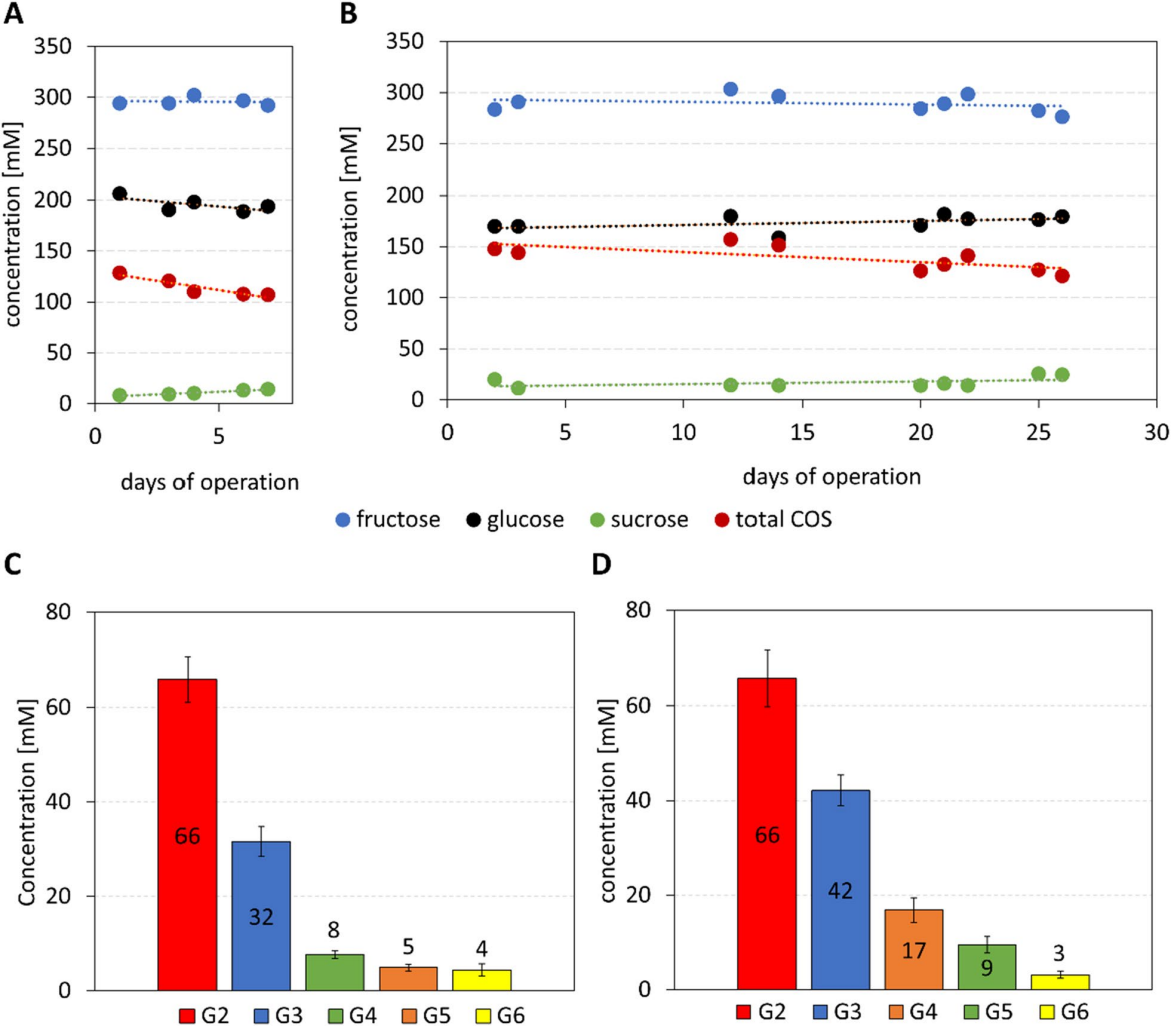
Stable continuous COS production for 7 (**A**) and 26 days (**B**) in operando and corresponding product mixtures broken down into the mean DP of COS (**C**, **D**). Standard deviations were calculated from 5 and 10 samples, respectively. For conditions, see Table 2, lines in bold font

Best conditions and results are marked in bold font and correspond to data shown in Fig. 6

Packed-bed reactor experiments performed with two different catalyst preparations (Fig. 6A, B) demonstrated continuous production for several days of a COS mixture of consistent composition. The G3–G6 content of the COS was largely constant over the reaction time in each experiment, but it changed by ~ 40% between the two experiments, apparently dependent on the catalyst preparation used (Fig. 6C, D; Table 2). One catalyst preparation was fresh (Fig. 6B, D) while the other had already been in use for ~ 2 weeks before (Fig. 6A, C). The G2 production was unaffected by the catalyst used (Fig. 6C, D). Stability of the CcCdP activity may thus limit the catalyst’s lifetime. In the experiment performed for 26 days (Fig. 6B), the concentration of total COS decreased by just ~ 10% from beginning to end, indicating excellent operational stability of the packed-bed reactor under the conditions used.

### Closing a recurrent mass balance gap

**Fig. 7:**
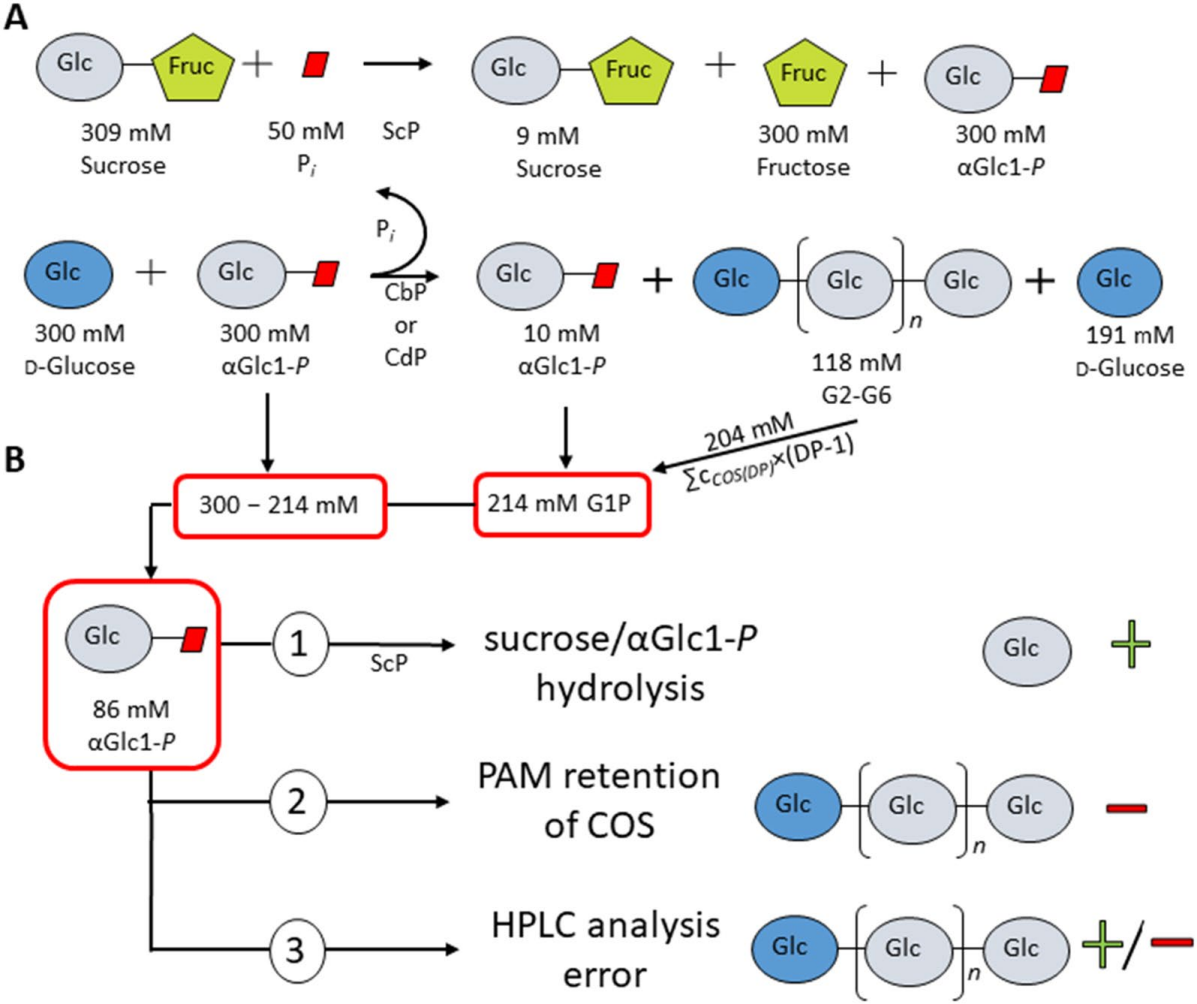
Analysis of mass balance in the enzymatic COS production in the packed-bed reactor. **A** Simplified reaction scheme and representative molar mass balances of the third day sample corresponding to Additional file 1: Table S4/experiment no. 4 in Table 2. *n* is 0 to 4. **B** Interacting reasons for the mass balance gap (86 mM αGlc1-*P*). For a discussion, see the text

The bottom-up synthesis of COS from sucrose and glucose involves the possibility of a complex distribution of glucosyl units into products (αGlc1-*P*, oligosaccharides of variable DP) that makes analysis of the reaction mass balance a non-trivial task. We were careful to ensure that the reported results meet the requirement of closed mass balance within limits of the experimental error. Indeed, data from (repetitive) batch reactions show excellent agreement between products released and substrate consumed (Additional file 1: Table S3), with deviations of maximally 4 to 7% that can be explained by the analytical error in HPLC especially for the longer oligosaccharides (see below). However, the continuous reactions involved a significantly larger gap (~ 28%; ~ 90 mM) in the glucosyl units mobilized from sucrose and effectively transferred into COS (G2 to G6; Additional file 1: Table S4). The gap was systematically positive, that is, more sucrose was converted than COS products were released. We explored possible reasons for the recurring mass balance gap based on considerations summarized in Fig. 7.

In the sample shown (Fig. 7A), sucrose (309 mM) was converted to 97% (9 mM left), implying a total of 300 mM glucosyl units mobilized in the form of αGlc1-*P*. Of these 300 mM, 290 mM were utilized for glycosylation reactions (10 mM αGlc1-*P* remaining) wherein 109 mM glucose were converted (191 mM remaining). The COS product mixture involved a molar content of glucosyl units transferred of 204 mM. Importantly, insoluble COS formation was negligible. Balance for αGlc1-*P* indicates 214 mM glucosyl equivalents present in the product mixture but 300 mM released from sucrose, resulting in a gap of 86 mM.

Partial hydrolysis of sucrose, αGlc1-*P* or both would explain usage of donor substrate larger than glucosyl transfer into COS product (G2 to G6; Fig. 7B). BaScP shows weak hydrolase activity towards sucrose and αGlc1-*P* (< 3% of the transfer activity to phosphate) [57]. The continuous reaction at 30 ℃ involved a 20-fold activity excess of BaScP over CuCbP and CcCdP (ratio: 10:0.6:0.7; Additional file 1: Table S2). Effectively all of the phosphate used (50 mM) was converted to αGlc1-*P* as glycosylation intermediate at steady state during the cascade conversion (Additional file 1: Fig. S2 top; SV 1.0 to 0.6 h^− 1^). With phosphate as the preferred nucleophile of the BaScP reaction depleted almost entirely, there was increased likelihood of hydrolysis to occur, in particular because sucrose was still present at a large concentration (≥ 200 mM). Similar in the batch reaction at 45 ℃ the intermittently accumulated αGlc1-*P* matched the total phosphate used (Additional file 1: Fig. S2 bottom; 0.5 to 6 hours; Additional file 1: Table S3). However, evidence that the glucose consumption was always well matched to the molar COS formation within a low error of ≤ 5% was not consistent with hydrolysis being a main factor. Additional glucose would be released in consequence of hydrolyses of sucrose and αGlc1-*P*.

Another possibility considered was that the longer-chain COS could experience diffusional restriction on their release from the PAM particles into the liquid bulk phase. With an estimated porosity (1 − volume of particles/total bed volume) of the packed-bed reactor of 0.31, solid-liquid partitioning of the COS dependent on their DP could explain a total COS concentration in the liquid phase lower than expected from the sucrose converted. As the effect of partitioning is dependent on porosity (i.e., the solid catalyst concentration used in the unit volume of reactor), it can be understood that the mass balance gap appeared only in the packed-bed reactor experiments (low porosity) and not in the batch reactions (high porosity, ~ 0.92; 8.1% catalyst loading). Partitioning effects would also affect the balance for glucose but evidently the COS involve their respective DP as “weighting factor”. As an example, error on the analytical determination of glucose by HPLC amounts to ~ 15 mM (5%). In the case of cellohexaose, this error would already be sufficient to explain a mass balance gap five times that of glucose (~ 75 mM). We therefore examined the analytical errors for each COS species based on HPLC measurements and authentic standards as reference. Errors were calculated from 3 randomly chosen samples from continuous production shown in Fig. 6A and C. Samples were measured using two individually prepared standard series in the same concentration range (G3: 50 to 5 mM, G4: 25 to 3 mM, G5: 10 to 1 mM, G6: 5 to 1 mM) to also account for weighing errors of individual COS species. The mean errors for G3 to G6 were ± 4.0 mM (12%), ± 1.2 mM (13%), ± 1.0 mM (18%) and ± 1.0 mM (17%), respectively (Additional file 1: Table S5). Considering these individual errors, the cumulated error on the determination of αGlc1-*P* used amounts to ~ 20 mM, equivalent to ~ 6% of total COS. It evidently increases as the relative distribution of individual oligosaccharides in total COS shifts towards the larger DPs.

In summary, the evidences just discussed lead to the suggestion that COS partitioning between the stationary solid phase (PAM particles) and the continuous liquid phase could affect (i.e., result in lowering of) the COS concentration measured in the product stream collected from the packed-bed reactor operated at steady state. Analytical error of HPLC measurements is important additionally. Both involve the oligosaccharide DP as weighting factor regarding the effect on mass balance. Concluding, therefore, the biocatalytic production of COS in the packed-bed reactor requires special attention to be paid to the mass balance. High-quality analytical data on the individual oligosaccharides formed are critical. Taking these points into account carefully, the results of the continuous reaction over 26 days (Fig. 6B) involved a presentable mass balance with low error of between 2% and 6% (Additional file 1: Table S6).

### Process options for continuous COS production

The fully continuous reaction in the packed-bed reactor is preferred for production. However, the repetitive batch reaction in a mixed vessel reactor with catalyst recycle might be an alternative option. The two modes of processing for COS production were compared based on the parameters summarized in Table 3. The product yield and composition of both reaction modes are comparable, however, the mass-based efficiency of the PAM catalyst in the packed bed reactor is only 24% that of the semi-permeabilized free cells in the mixed vessel reactor. In addition to cell encapsulation, which lowers the activity of the catalyst by 57%, the temperature reduction from 45 °C to 30 °C also leads to a decrease of ~ 65%. However, advantages of the packed bed reactor over the mixed vessel reactor are the ease and effortlessness of handling during the production; the controllability of reaction conditions and parameters; the consistency of the product quality in long-term production (one month); and the avoidance of catalyst separation from the product mixture. Moreover, when scaling-up the mixed vessel reaction, the problem of damage to the particles or free cells at high agitation speeds must be considered as this may cause difficulties in their recovery and re-use [6]. Two important challenges in scaling-up the continuous COS production are a scale-dependent increase of time constants and a limited bed height due to the risk of pressure drop across the bed [6]. However, crucial for scale-up is a good understanding of the biocatalyst and the chemical and physical environment in the reactor [6], which was successfully achieved in this study.

**Table 3:** Key performance metrics of repetitive batch and continuous production of soluble COS (G2 to G6) with free (semi-permeabilized) and PAM immobilized cells, respectively

Performance metric	Repetitive batch production/mixed vessel reactor	Continuous production/packed-bed reactor
	Semi-permeabilized whole-cell catalysts (45 °C)	PAM immobilized whole-cell catalysts (30 °C)
Operation time [h]	30 (5 cycles of 6 h each)	624
COS [g/L]	68	66
STY [g/L/h]	11	6.6 (24%)^a^
Product solution [mL]	1250	2250
Total COS [g]	85	150
COS composition [mM]	G2–G6: 70, 49, 22, 5, 1	G2–G6: 66, 42, 17, 9, 3
Cell dry mass (CDM) [g]	0.75	1.85
TTN [g_COS_/g_CDW_]	113	81
Normalized COS yield [gCOS/gCDM/h]	3.8	0.13

^a^ Percent mass-based efficiency of the PAM catalyst in the packed-bed reactor compared to the mixed vessel reactor used for repetitive batch production

## Conclusion

Continuous production of soluble COS was shown with an immobilized whole cell catalyst that promotes a three-enzyme cascade transformation of sucrose and glucose. The COS were obtained at a concentration of ~ 70 g/L in a consistent composition of individual oligosaccharides. The continuous process was operated stably for ~ 1 month, suggesting a robust process technology for biocatalytic production at controlled quality. Formation of insoluble COS was successfully avoided. The production of the COS shown here can keep up with the efficient production of other non-digestible oligosaccharides, such as galacto-oligosaccharides (GOS; ~ 350 g/L) or fructo-oligosaccharides (FOS; ~ 100 g/L) [58, 59]. Collectively, cascade biocatalysis in immobilized whole cells is shown to be feasible for continuous production in a packed-bed reactor. The continuous reaction is instrumental to maintain the quality of the product mixture in long-term operation [6, 13, 60].

## Methods

### Cell preparation

The previously designed plasmid expression vector pPOLY-2 (https://www.addgene.org/179276/) was used for the tricistronic coexpression of BaScP (GenBank identifier AF543301.1), CuCbP (GenBank identifier AAQ20920.1) and CcCdP (GenBank identifier CDZ24361.1). Further details on the plasmid design can be found in our previous publication [39]. pPOLY-2 was transformed into *E. coli* BL21(DE3)*agp*^-^ (obtained from Tom Desmet, Ghent University, BEL) using electroporation [61]. Usage of an *agp*-deficient *E. coli* strain prevents degradation of αGlc1-*P* in whole-cell conversions. *E. coli* strains were grown at 37 ℃ in LB-medium (5 g/L NaCl, 5 g/L yeast extract, 10 g/L peptone from casein) in baffled shake flasks containing 100 mg/L ampicillin. Main cultures, 250 mL medium in a 1 L flask, were inoculated with cells from an overnight preculture to an optical density at 600 nm (OD_600_) of 0.03. Biomass was grown to an OD_600_ of 0.8–1.0 and expression was induced with 1 mM IPTG. Coexpression was performed for ~ 18 h at 25 °C and 110 rpm in incubation shaker CERTOMAT BS-1 (Sartorius, Göttingen, DE). The OD_600_ was measured spectrophotometrically (DU^®^ 800 UV/Vis Spectrophotometer, Beckman Coulter, Brea, CA, USA). Centrifuged cells (20 min, 4 °C, 4.4 krcf, Ultracentrifuge Sorvall RC-5B Superspeed, Thermo Fisher Scientific Inc., Waltham, MA, USA) were resuspended in 6 mL 50 mM MES buffer, pH 7.0, per ~ 1 g cell wet weight, and aliquoted (~ 10–15 mL portions).

### Cell weight determination

2 mL of cell suspension (in 50 mM MES buffer) were transferred in a preweighed 2 mL Eppendorf tube and centrifuged (20 min, 21.1 krcf, Centrifuge Eppendorf 5424 R, Eppendorf, Hamburg, DE). The supernatant was thoroughly removed. After one washing step the cell wet weight (CWW) was determined with an analytical balance. The cell pellet was dried overnight at 70 ℃ and the cell dry weight was determined. Determination was done three times from the same cell suspension and revealed a variation factor of ~ 5% (~ 25 mg_CDW_/mL).

### Cell permeabilization and PAM immobilization

*Escherichia coli* cells (10–15 mL of 25 g_CDW_/L, 50 mM MES buffer) were frozen to − 70 °C and thawed after 12 h or more. The permeabilized cells were used directly in reactions (free cells) or were immobilized in PAM. For PAM immobilization, the pelleted cells (10 min, 21.1 krcf, Centrifuge Eppendorf 5424 R, Eppendorf) were suspended with magnetic stirring at 300 rpm in 20 mL of 100 mM MES buffer (pH 7.0). The cell loading was varied at 0.2, 0.4, and 0.6 g wet cells/mL, equivalent to 50, 100, and 150 mg cell dry weight/mL or 37, 74 and 111 mg cell dry weight/g PAM material. Acrylamide (97%; Sigma Aldrich/Merck) was added to 0.1875 g/mL. Then, 10 mg/mL N,N′-methylenebis(acrylamide) (Carl Roth, Karlsruhe, DE) and a 5 vol% 3-(dimethylamino)propionitrile (98%; Sigma-Aldrich) solution were admixed (each at 0.125 mL/mL cell suspension) as cross-linking agent and polymerization accelerator, respectively. Polymerization was started with 2.5 wt% potassium persulfate (99%; Sigma Aldrich/Merck) solution and was performed in an ice batch for 30 min. The obtained PAM material was cut with a scalpel into 3–5 mm particles (Additional file 1: Fig. S1A) and further shredded into smaller particles with a hand blender for some seconds. The resulting PAM particles (Additional file 1: Fig. S1C) were sieved to a mesh size of 0.25–2.00 mm and washed thoroughly with deionized water on the sieves. Unless mentioned, the 0.25–2.00 mm particles were used. Prior to use in conversion studies, the PAM particles were washed at 21 °C with 50 mM MES buffer in stirred suspension (100 rpm) or in the packed bed under continuous flow.

### Enzyme activity measurements

General procedure: Enzyme activities were measured in discontinuous assays done at 30 and 45 °C, as indicated under Results. Free and PAM-immobilized cells were used. Typically, 0.5–5 mg cell dry mass in suspension or immobilized cells with a cell loading of 37 mg_CDW_/g_PAM_ and a particle size of 0.25 to 2.00 mm were mixed into the reaction mixture with a total volume of 2 mL. Incubations were in 2 mL agitated at 900 rpm. Samples (200 µL) were taken every 2–5 min up to 30 min. The sample was heated for 2 min at 100 °C.

BaScP: Reaction was done with 250 mM sucrose and 50 mM phosphate in 50 mM MES buffer, pH 7.0. The αGlc1-*P* was converted by phosphoglucomutase from rabbit muscle (3 U/mL, Sigma-Aldrich/Merck) and NAD^+^-dependent d-glucose-6-phosphate dehydrogenase from *Leuconostoc mesenteroides* (3.4 U/mL, Sigma-Aldrich/Merck) to gluconolactone 6-*P*. The equimolar NADH was monitored spectrophotometrically at 340 nm (DU^®^ 800 UV/Vis Spectrophotometer Beckman Coulter).

CuCbP: Cellobiose synthesis was done from 50 mM glucose and 50 mM αGlc1-*P* (Sigma-Aldrich/Merck) in 50 mM MES buffer, pH 7.0. Phosphate release from αGlc1-*P* was measured using the colorimetric assay of Saheki et al. [62]. To avoid interference with CcCdP activity, the assay was performed with another catalyst that lacked CcCdP but provided the same CuCbP activity as the current cell catalyst (3.0 and 2.8 U/mg, respectively; https://www.addgene.org/155165/) [39, 63].

CcCdP: The reaction was performed with 50 mM *p*-nitrophenyl β-D-cellobioside (*p*NP-G2; CarboSynth, Compton, Berkshire, UK), 50 mM αGlc1-*P*, 50 mM MES buffer, pH 7.0. Phosphate was measured using the assay of Saheki et al. [62]. *p*NP-G2 was used to avoid interference with CuCbP activity. Previously, activities of purified CcCdP were compared on both, cellobiose and *p*NP-G2 [32, 39]. Activities on *p*NP-G2 were converted to activities on cellobiose by the factors 0.84 and 0.52 for measurements performed at 45 °C and 30 °C, respectively [39].

For all measurements one unit (U) of activity is the enzyme amount producing 1 µmol αGlc1-*P*/min (BaScP) or 1 µmol phosphate/min (CuCbP and CcCdP) under the conditions employed.

### Batch COS bioproduction in agitated vessel with catalyst recycling

First batch reactions were performed in 100 mL borosilicate glass bottles (50 mL reaction volume) and repetitive batch productions in 500 mL bottles (250 mL), both equipped with Rotilabo^®^ magnetic stirrer bars (25 × 8 mm, Carl Roth GmbH). The substrate solutions contained 500 mM sucrose, 200 mM glucose, 50 mM phosphate, 50 mM MES buffer, pH of 7.0. Reactions were started with free or immobilized cells (0.25–2.00 mm particles with a cell loading of 37 mg_CDW_/g_PAM_), resulting in concentrations of 3 g_CDW_/L reaction volume. Reaction temperatures were 45 °C and 35 °C and were maintained by an incubation shaker (CERTOMAT BS-1, Sartorius). Reactions were performed on a Variomag^®^ Multi-Magnetic Stirrer (Thermo Fisher Scientific Inc.; rpm 300) for 26 h, 6 or 8 h, respectively. 1 mL of sample were taken each hour or after each batch and centrifuged (10 min 21.1 krcf, 21 °C for 45 min, Centrifuge Eppendorf 5424 R, Eppendorf). The supernatant was heated to 100 ℃ to inactivate residual enzymes/biomass. The samples were stored at room temperature until HPLC measurement. Separation of the catalysts from the product solution after each batch cycle was performed either by centrifugation of the free cell catalyst (20 min, 21 °C, 4.4 krcf, Ultracentrifuge Sorvall RC-5B Superspeed, Thermo Fisher Scientific Inc.) or by sieving of the immobilized cells (mesh size of 180 μm). The heat-treated (30 min in a boiling water bath) and centrifuged (45 min, 21 °C, 17.0 krcf) product solutions were kept at room temperature to avoid temperature-induced precipitation of COS, until combined after the five repeated batch cycles. The catalysts were stored at 4 °C overnight until the next batch was performed.

### Continuous COS bioproduction in a packed-bed reactor

PAM particles (size range in mm: 0.25–2.00 and ~ 3–5 mm) were packed by hand into XK26/20 columns (ID 26 mm, GE Healthcare, Chicago, IL, USA) and thoroughly flushed overnight with 50 mM MES buffer, pH 7.0 at room temperature. The reactor bed volume was 40 mL, when particles of ~ 3–5 mm were used, and 36 mL, when particles of 0.25–2.00 mm were used. The column temperature was controlled at 45 °C or 30 °C from a circulating water bath connected to the column’s jacket. A Smartline Pump 1000 (Knauer, Berlin, DE) delivered feed from the substrate solution. The feed flow rate (space velocity), the composition of the substrate and the cell loading of particles varied as indicated in the results. Samples (1 mL) were taken at the reactor outlet, heated (5 min, 99 °C), centrifuged (10 min, 21.1 krcf, 21 °C for 45 min, Centrifuge Eppendorf 5424 R, Eppendorf) and analyzed by HPLC.

The space velocity, space time yield and total turnover number were calculated with equations stated in Table 4.

**Table 4:** Equations for calculating important performance metrics of the continuous production

Performance parameter	Space velocity (SV; h^− 1^)	Space-time yield (STY; g/L/h)	Total turnover number (TTN; g_COS_/g_CDW_)
Equation	$SV=\frac{\nu }{V}=\frac{1}{\tau }$	$STY={c}_{G2-G6}\times SV$	$TTN=\frac{SV\times V\times t\times {c}_{G2-G6}}{m}$

*v* was the flow rate, *V* was the reactor volume, τ was the residence time, *c* was the sum of the product concentrations (g/L) of G2 to G6, *t* was the operation time in hours, *m* was the mass of applied dry cells.

### HPLC analysis of reaction compounds

HPLC measurements were performed with a Merck Hitachi L-7100 system (Merck, Darmstadt, DE) equipped with an autosampler (L-7250) and a refractive index detector (L-7490). Separation of sucrose, glucose, fructose and cellobiose was performed with an YMC-Pack Polyamine II/S-5 μm/12 nm column (250 mm ⋅ 4.6 mm; YMC Co., Ltd., Shimogyo-ku, Kyoto, JP). Additionally, a guard column (20 mm × 4.0 mm; YMC Co., Ltd.) was installed. Elution was performed isocratically with an acetonitrile-water mixture (75:25, v:v) at a flow rate of 1 mL/min. Measurements were performed at room temperature, the injection volume per sample was set to 20 µL and the running time was 35 min. Soluble COS (DP2 to DP6) were quantified by a Luna 5 μm NH2 column (100 Å, 250 × 4.6 mm, Phenomenex, Aschaffenburg, DE) operated at 40 °C. Acetonitrile-water (70:30, v:v) was used as eluent at a flow rate of 1.5 mL/min and a running time of 15 min.

Representative chromatograms of product solutions measured by the stated HPLC methods are shown in Additional file 1: Fig. S3. Refractive index detected peaks were analyzed using the software Chromeleon Chromatography Data System (Thermo Fischer Scientific Inc.). Calibration was done with reagent-grade COS standards ranging from DP2 to DP6 (> 95%; > 90% for DP4) and were from Megazyme Ltd. (Wicklow, IRL).

αGlc1-*P* concentrations in product solutions from batch and continuous productions were measured with the assay used to determine BaScP activity (“Enzyme activity measurements” section).

## Supplementary Information

**Additional file 1**: **Figure S1.** Limitations of continuous COS production in a packed-bed reactor. (A) 3 to 5 mm particles before (left) and after COS production at 45 °C showing a whitish discoloration (right); (B) 0.25–2.00 mm sized particles in the swollen (30 °C; left) and shrunken (45 °C; right) state; (C) intact packed-bed reactor before (left) and broken reactor after (right) COS production. **Figure S2.** Free (left y-axis) and converted (COS-integrated; right y-axis) αGlc1-*P* in continuous (top) and batch (bottom) reaction with immobilized cells (37 and 74 mg_CDW_/g_PAM_, respectively). **Figure S3.** HPLC chromatograms of representative product solution (1st day of long-term continuous COS production). Mono- and disaccharides were measured with an YMC-Pack Polyamine II/S-5 µm/12 nm column (A) and oligosaccharides (cello-oligosaccharides G2 to G6) were measured with a Luna5 µm NH2 column (B). **Table S1.** Molar concentrations and overall activities of repetitive batch reactions with immobilized cell catalysts at 35 °C and 45 °C. **Table S2.** Activity ratio of enzymes in PAM-immobilized whole cells, measured at 30 °C. All activities were measured in synthesis direction (see “Enzyme activity measurements” section). **Table S3.** Representative molar mass balances of batch reaction (6 h) with immobilized cells (Fig. 3). **Table S4.** Representative molar mass balances of continuous reactionwith immobilized cells (Fig. 5A, sample from the 3rd day). **Table S5.** HPLC measurements of single COS species of three samples from continuous production shown in Figure 6A and C. Samples were measured with two separately prepared standard series (1 and 2) in the same concentration range (G3:50 to 5 mM, G4: 25 to 3 mM, G5: 10 to 1 mM, G6: 5 to 1 mM). **Table S6.** Representative molar mass balances (measured with a new LUNA-column) of continuous reaction with immobilized cells (Fig. 5B, sample from the 22nd day).

## Data Availability

The datasets supporting the conclusions of this article are available in the zenodo repository, 10.5281/zenodo.7184673.

## Abbreviations

αGlc1-*P*: α-d-Glucose-1-phosphate
BaScP: Sucrose phosphorylase from *Bifidobacteium adolescentis*
CcCdP: Cellodextrin phosphorylase from *Clostridium cellulosi*
CDW: Cell dry weight
COS: Cello-oligosaccharides
CuCbP: Cellobiose phosphorylase from *Cellulomonas uda*
DP: Degree of polymerization
FOS: Fructo-oligosaccharides
GOS: Galacto-oligosaccharides
MES: 2-(*N-*morpholino)ethanesulfonic acid
PAM: Polyacrylamide
***p***NP-G2: *p-*nitrophenyl β-d-cellobioside
pPOLY: Tricistronic co-expression plasmid
STY: Space-time yield
SV: Space velocity
TTN: Total turnover number
