# Supplementary material for: Continuous process technology for bottom-up synthesis of soluble cello-oligosaccharides by immobilized cells co-expressing three saccharide phosphorylases
Source: Microb Cell Fact. 2022 Dec 19;21:265. doi: 10.1186/s12934-022-01984-1 (PMC9764710; doi:10.1186/s12934-022-01984-1)
Supplement: Supplementary file 1 — Additional file 1: Figure S1. Limitations of continuous COS production in a packed-bed reactor. (A) 3 to 5 mm particles before (left) and after COS production at 45 °C showing a whitish discoloration (right); (B) 0.25–2.00 mm sized particles in the swollen (30 °C; left) and shrunken (45 °C; right) state; (C) intact packed-bed reactor before (left) and broken reactor after (right) COS production. Figure S2. Free (left y-axis) and converted (COS-integrated; right y-axis) αGlc1-P in continuous (top) and batch (bottom) reaction with immobilized cells (37 and 74 mgCDW/gPAM, respectively). Figure S3. HPLC chromatograms of representative product solution (1st day of long-term continuous COS production). Mono- and disaccharides were measured with an YMC-Pack Polyamine II/S-5 µm/12 nm column (A) and oligosaccharides (cello-oligosaccharides G2 to G6) were measured with a Luna5 µm NH2 column (B). Table S1. Molar concentrations and overall activities of repetitive batch reactions with immobilized cell catalysts at 35 °C and 45 °C. Table S2. Activity ratio of enzymes in PAM-immobilized whole cells, measured at 30 °C. All activities were measured in synthesis direction (see “Enzyme activity measurements” section). Table S3. Representative molar mass balances of batch reaction (6 h) with immobilized cells (Fig. 3). Table S4. Representative molar mass balances of continuous reactionwith immobilized cells (Fig. 5A, sample from the 3rd day). Table S5. HPLC measurements of single COS species of three samples from continuous production shown in Figure 6A and C. Samples were measured with two separately prepared standard series (1 and 2) in the same concentration range (G3:50 to 5 mM, G4: 25 to 3 mM, G5: 10 to 1 mM, G6: 5 to 1 mM). Table S6. Representative molar mass balances (measured with a new LUNA-column) of continuous reaction with immobilized cells (Fig. 5B, sample from the 22nd day). [file 12934_2022_1984_MOESM1_ESM.pdf]

Continuous process technology for bottom-up synthesis of soluble cello-oligosaccharides by immobilized cells co-expressing three saccharide phosphorylases

Katharina N. Schwaiger[1], Bernd Nidetzky[1,2]

[1]acib - Austrian Centre of Industrial Biotechnology, Krenngasse 37, 8010, Graz, Austria

[2]Institute of Biotechnology and Biochemical Engineering, Graz University of Technology, NAWI Graz, Graz, Austria

Correspondence: Bernd Nidetzky, Institute of Biotechnology and Biochemical Engineering, TU Graz, NAWI Graz, Petersgasse 12, 8010 Graz, Austria

E-mail: bernd.nidetzky@tugraz.at

Keywords: Cello-oligosaccharides, multi-enzymatic cascade, immobilized whole-cell catalyst, repetitive batch cycles, continuous flow bioproduction

Content

Figure S1………2

Figure S2………3

Figure S3………4

Table S1, S2……5

Table S3………6

Table S4 ………7

Table S5 ………8

Table S6 ………9

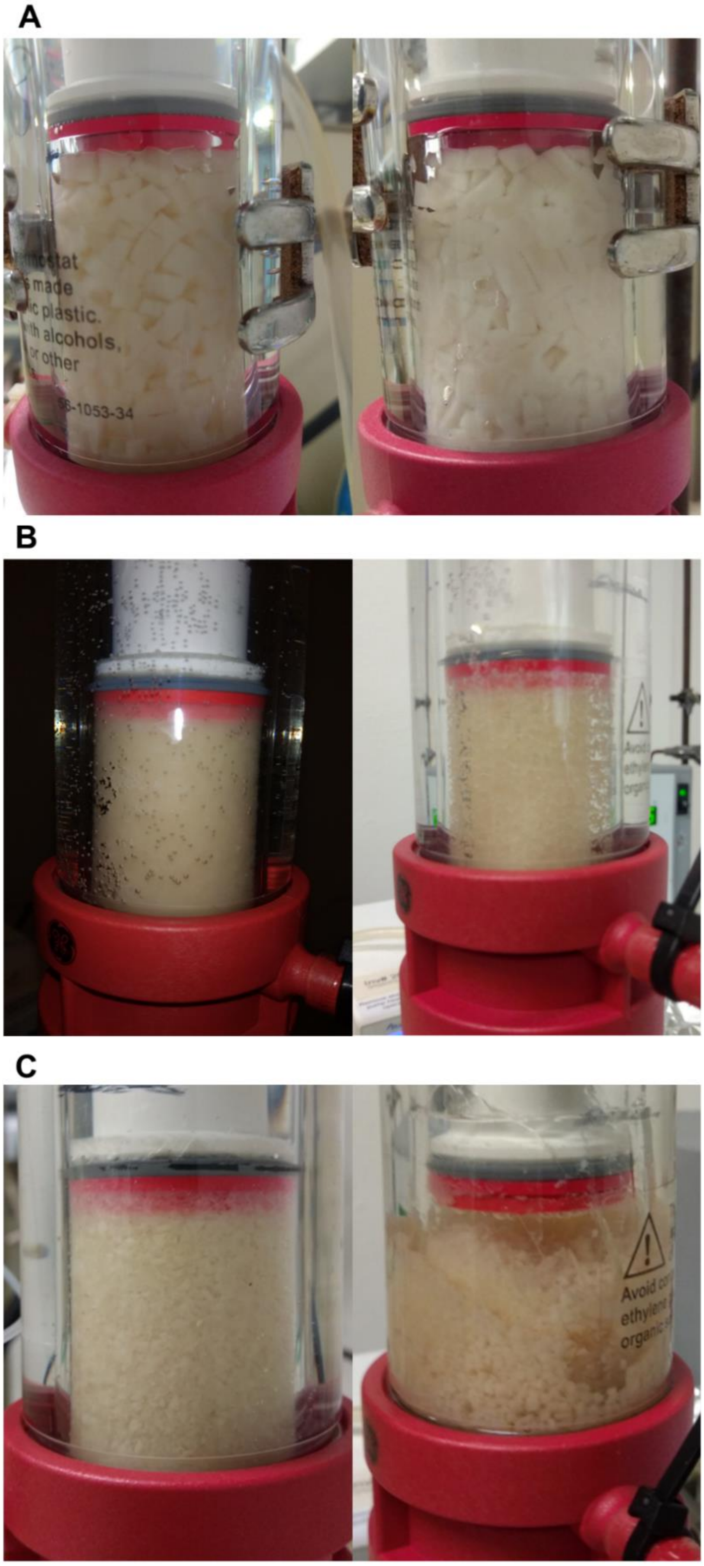

Figure S1: Limitations of continuous COS production in a packed-bed reactor. (A) 3 to 5 mm particles before (left) and after COS production at 45 °C showing a whitish discoloration (right); (B) 0.25-2.00 mm sized particles in the swollen (30 °C; left) and shrunken (45 °C; right) state; (C) intact packed-bed reactor before (left) and broken reactor after (right) COS production.

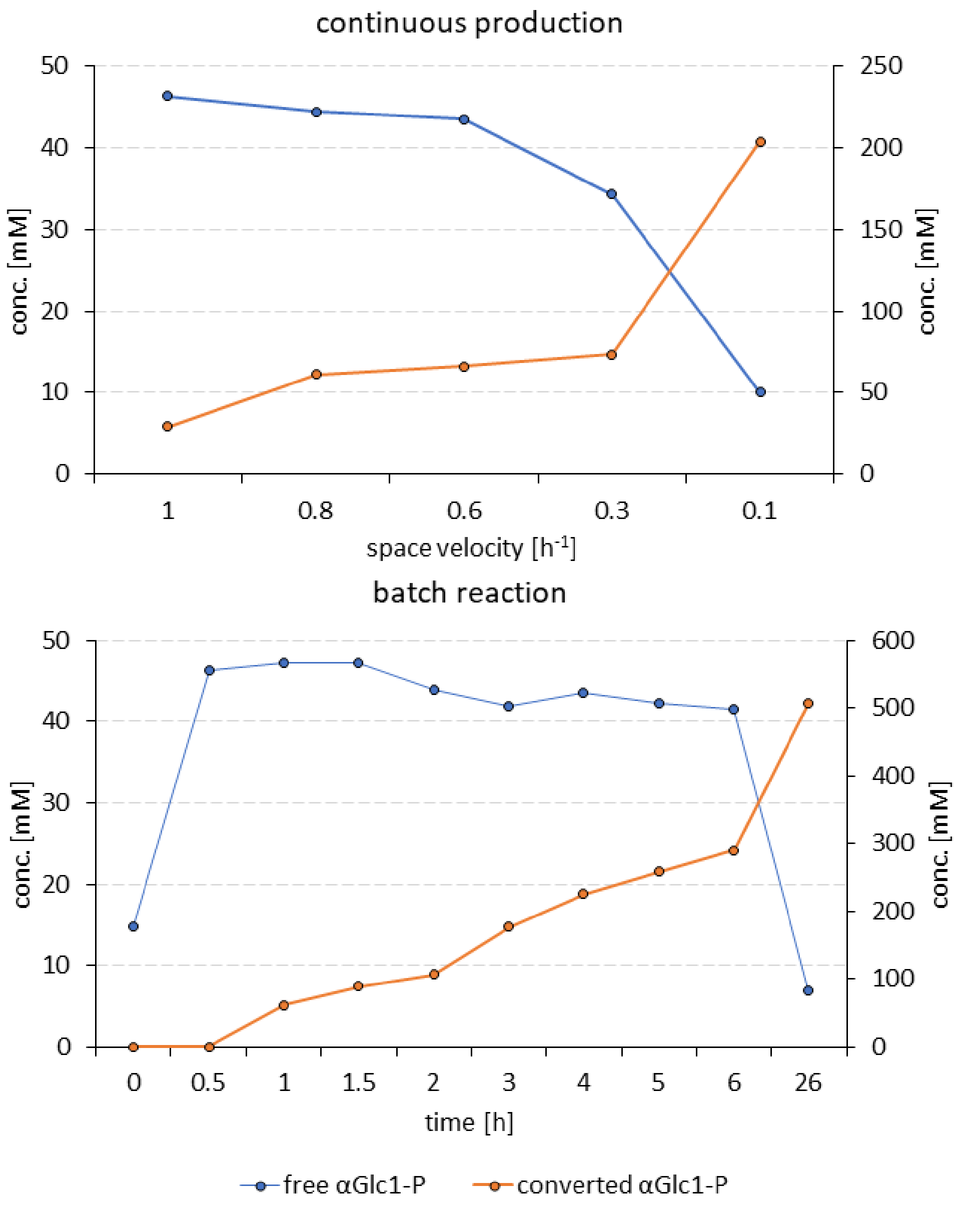

Figure S2: Free (left y-axis) and converted (COS-integrated; right y-axis) αGlc1-*P* in continuous (top) and batch (bottom) reaction with immobilized cells (37 and 74 mg_{CDW}/g_{PAM}, respectively).

A

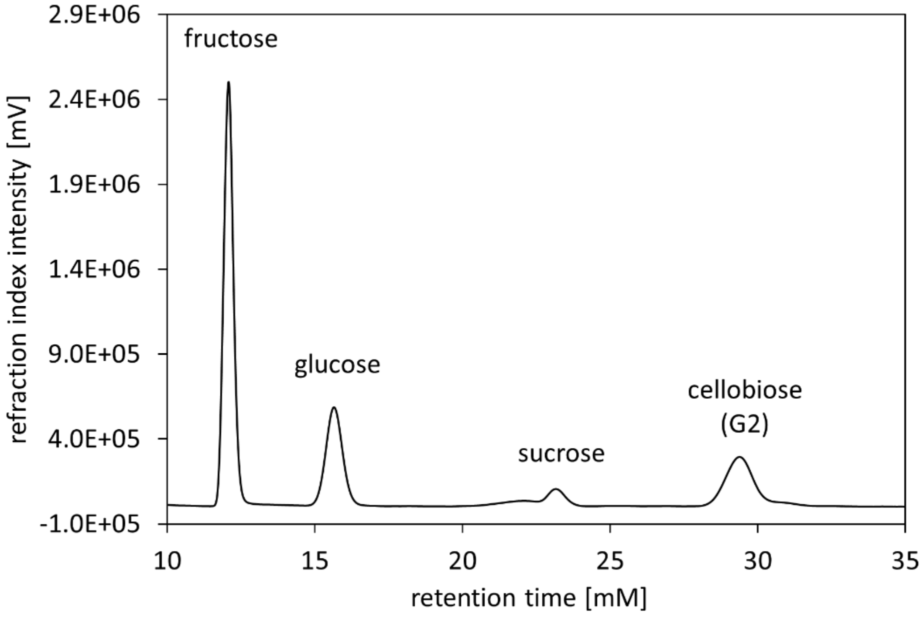

B

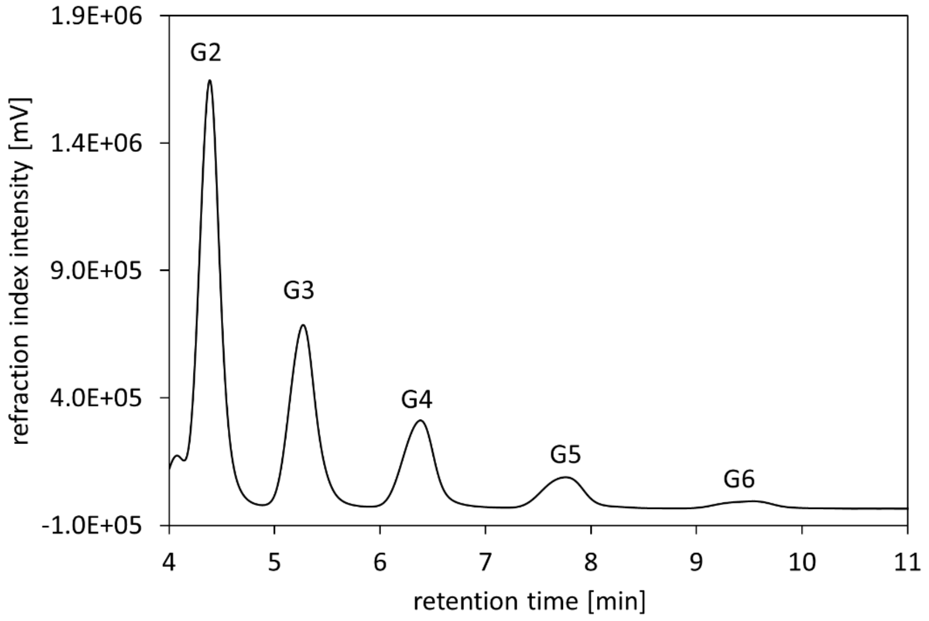

Figure S3: HPLC chromatograms of representative product solution (1st day of long-term continuous COS production). Mono- and disaccharides were measured with an YMC-Pack Polyamine II/S-5 µm/12 nm column (A) and oligosaccharides (cello-oligosaccharides G2 to G6) were measured with a Luna 5 µm NH2 column (B).

Table S1: Molar concentrations and overall activities of repetitive batch reactions with immobilized cell catalysts at 35 °C and 45 °C.

35 °C 8h	*G2 [mM]*	*G3 [mM]*	*G4 [mM]*	*G5 [mM]*	*G6 [mM]*	*total COS [mM]*	*incorp. αGlc1-P [mM]*	*U/gCDW*
batch 1	43.4	40.2	15.7	5.5	2.8	107.6	207.1	143.9
batch 2	14.5	32.6	14.7	6.7	2.4	70.9	162.5	112.9
batch 3	16.3	30.8	12.8	6.1	2.4	68.5	153.0	106.3
batch 4	15.4	33.0	13.2	6.6	2.8	70.9	161.2	111.9
batch 5	15.3	30.6	10.4	4.4	0.9	61.6	129.8	90.1
mean	**21.0**	**33.5**	**13.4**	**5.9**	**2.3**	**75.9**	**162.7**	**113.0**
45 °C 6h	*G2 [mM]*	*G3 [mM]*	*G4 [mM]*	*G5 [mM]*	*G6 [mM]*	*total COS [mM]*	*incorp. αGlc1-P [mM]*	*U/gCDW*
batch 1	40.3	40.0	29.5	16.6	5.6	132.1	303.4	281.0
batch 2	17.2	21.0	17.5	11.4	3.9	71.0	176.7	163.6
batch 3	13.0	17.8	12.0	10.0	4.5	57.3	147.2	136.3
batch 4	12.0	16.0	10.0	9.0	5.5	52.5	137.5	127.3
batch 5	9.7	12.4	9.1	8.3	8.2	47.7	136.1	126.0
mean	**18.4**	**21.4**	**15.6**	**11.1**	**5.5**	**72.1**	**900.9**	**166.8**

Table S2: Activity ratio of enzymes in PAM-immobilized whole cells, measured at 30 °C. All activities were measured in synthesis direction (see Methods Part "Enzyme activity measurements").

	**[U/gCDW]**	**ratio**
BaScP	820 ± 30	10
CuCbP	52 ± 6	0.6
CcCdP	63 ± 6	0.7

Table S3: Representative molar mass balances of batch reaction (6 hours) with immobilized cells (Figure 3)

	Glu	*total COS**	*COS + Glu*	***Suc***	*αGlc1-P incorp.*	*αGlc1-P not incorp*	*Suc + αGlc1-P*	***Suc***	*Fru*	*Suc + Fru*
substrate [mM]	228			523				523		
product (6h) [mM]	84	152[a]	237	227	289[b]	41[c]	558	227	277	50 4
conversion	63%			57%				57%		
yield		67%			55%				53%	
gap[d] [mM]			8				34			19
gap[d]			**4%**				**7%**			**4%**

[a]total COS concentration was calculated by the sum of single COS species (G2 to G6; 62, 54, 26, 9, 1 mM respectively)

[b]αGlc1-*P* in product solution measured by αGlc1-*P* spectrophotometric assay (see Methods-Part "Enzyme activity measurements"))

[c]COS-incorporated αGlc1-*P* derived glucose-moieties. The sum of glucosyl units was obtained as ∑cCOS(DP) × (DP-1), where cCOS(DP) is the concentration of the individual soluble COS of a certain DP (DP 2 to 6 corresponds to G2 to G6).

[d]molar or percentage gap between substrate and product.

Table S4: Representative molar mass balances of **continuous reaction** with immobilized cells (Figure 5A, sample from the 3rd day)

	Glu	*total COS**	*COS + Glu*	***Suc***	*αGlc1-P incorp.*	*αGlc1-P not incorp*	*Suc + αGlc1-P*	***Suc***	*Fru*	*Suc + Fru*
substrate [mM]	312			309				309		
product (3rd day) [mM]	191	118[a]	308	9	204[b]	10[c]	223	9	294	303
conversion	39%			97%				97%		
yield		38%			66%				95%	
gap[d] [mM]			4				86			5
gap[d]			**1%**				**28%**			**2%**

[a]total COS concentration was calculated by the sum of single COS species (G2 to G6; 64, 35, 8, 6, 4 mM respectively)

For remaining endnotes see table S3

Table S5: HPLC measurements of single COS species of three samples from continuous production shown in Figure 6A and C. Samples were measured with two separately prepared standard series (1 and 2) in the same concentration range (G3: 50 to 5 mM, G4: 25 to 3 mM, G5: 10 to 1 mM, G6: 5 to 1 mM).

		**sample 1**	**sample 2**	**sample 3**	**mean error**
**G3**	1 [mM]	31.9	33.6	34.6	
	2 [mM]	34.7	29.6	29.3	
	deviation	2.8	4.0	5.2	**4.0**
	mean	33.3	31.6	31.9	
	error	*8%*	*13%*	*16%*	**12%**
**G4**	1 [mM]	10.8	7.2	7.9	
	2 [mM]	8.4	7.5	7.0	
	deviation	2.4	0.2	0.9	**1.2**
	mean	9.6	7.3	7.4	
	error	*25%*	*3%*	*12%*	**13%**
**G5**	1 [mM]	7.0	4.5	6.2	
	2 [mM]	6.1	5.6	5.1	
	deviation	1.0	1.0	1.1	**1.0**
	mean	6.5	5.1	5.6	
	error	*15%*	*21%*	*19%*	**18%**
**G6**	1 [mM]	7.0	5.0	5.2	
	2 [mM]	6.2	6.3	6.1	
	deviation	0.9	1.3	0.9	**1.0**
	mean	6.6	5.7	5.6	
	error	*13%*	*23%*	*16%*	**17%**
**total COS**	1 [mM]	56.7	50.4	53.8	
	2 [mM]	55.3	48.9	47.5	
	deviation	1.4	1.5	6.3	**3.1**
	mean	56.0	49.7	50.6	
	error	*3%*	*3%*	*12%*	**6%**

Table S6: Representative molar mass balances (measured with a new LUNA-column) of **continuous reaction** with immobilized cells (Figure 5B, sample from the 22nd day)

	Glu	*total COS**	*COS + Glu*	***Suc***	*αGlc1-P incorp.*	*αGlc1-P not incorp*	*Suc + αGlc1-P*	***Suc***	*Fru*	*Suc + Fru*
substrate [mM]	304			306				306		
product (22nd day) [mM]	177	141[a]	318	14	265[b]	10[c]	289	14	298	312
conversion	42%			95%				95%		
yield		46%			86%				97%	
gap[d] [mM]			14				17			6
gap[d]			**5%**				**6%**			**2%**

[a]total COS concentration was calculated by the sum of single COS species (G2 to G6; 67, 43, 17, 10, 4 mM respectively)

For remaining endnotes see table S3
